# VibeTube: a code for accurate extraction of CNTs’ chirality from TEM images

**DOI:** 10.1080/14686996.2026.2705816

**Published:** 2026-07-20

**Authors:** Daiming Tang, Gaurav Raj, Emmanuel Picheau, Shaaista Hasan, Peng-Xiang Hou, Chang Liu

**Affiliations:** a Research Center for Materials Nanoarchitectonics (MANA), National Institute for Materials Science (NIMS), Tsukuba, Japan; b Institute of Pure and Applied Sciences, University of Tsukuba, Tsukuba, Japan; c Department of Metallurgical and Materials Engineering, Indian Institute of Technology Roorkee, Roorkee, India; d Shenyang National Laboratory for Materials Science, Institute of Metal Research (IMR), Chinese Academy of Sciences, Shenyang, PR China; e School of Materials Science and Engineering, University of Science and Technology of China, Hefei, PR China

**Keywords:** Carbon nanotube, chirality, diameter, transmission electron microscope, fast Fourier transform, AI for science, Python code

## Abstract

Accurate determination of the chiral indices of carbon nanotubes (CNTs) is crucial for their controllable synthesis and practical applications. Transmission electron microscopy (TEM) combined with electron diffraction has been one of the most reliable methods for characterizing the chiral indices of CNTs. However, it is still a challenge to analyze TEM images to extract chirality with high efficiency and accuracy, especially for nanotubes with a diameter larger than 2 nanometers. In this work, a Python code is developed with assistance from an artificial intelligence model (Claude Code) for the auto-extraction of CNTs’ chirality from TEM images. Precise and reliable measurement of the diameter was realized by using a new method to determine the average distance between the bright and dark fringes. Symmetry and geometry-based rules enabled measurements of layer lines in the Fourier transformation of TEM images with a sub-pixel resolution. As a result, an accuracy higher than 90% for CNTs with diameters ranging from 0.5 nm to 3.0 nm was achieved. High robustness has been validated by experimentally analyzing the chiralities of CNTs grown by a floating catalyst chemical vapor deposition method. The open-source codes and tools will be valuable for the research community to quantitatively identify the chirality distribution of CNTs with high efficiency.

## IMPACT STATEMENT

Developed with an AI co-scientist (Claude Code), ‘VibeTube’ is an open-source, interpretable, and physics-based Python code for automated, accurate, and robust extraction of carbon nanotube chiralities from TEM images.

## GRAPHICAL ABSTRACT

**Figure:**
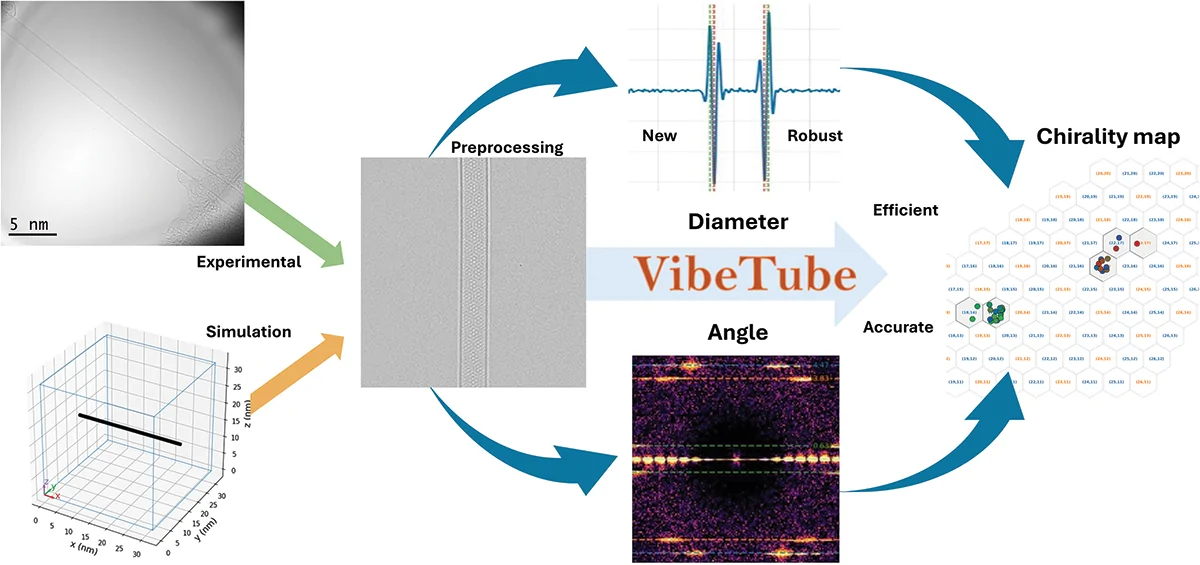

## Introduction

Electronic properties of carbon nanotubes (CNTs) are determined by the so-called chirality, that is the chiral vector associated with quantization along the circumference. Depending on the chiral indices (n, m), CNTs can be metallic or semiconducting [1]. Therefore, it is critical to accurately determine the chirality for understanding the precise chirality-related fundamental properties and their practical applications in electronics and optics [2–4].

Spectroscopic methods have been developed for characterization of CNTs’ chiralities, including resonant Raman scattering [5,6], photoluminescence (PL) [7,8], and Rayleigh scattering [9,10]. Optical spectroscopic techniques have advantages of being non-destructive, high throughput, high sensitivity to individual nanotubes, and low cost. Microscopic techniques were applied to determine the atomic structure of CNTs. By using scanning tunneling microscopy (STM) combined with scanning tunneling spectroscopy (STS), not only atomic configurations but also electronic structures of individual nanotubes including nanotube junctions were obtained [11]. Transmission electron microscope (TEM) has been one of the most powerful instruments to characterize the atomic structure and properties of CNTs. In 1991, Iijima discovered these small graphitic tubes and identified their helical structures by electron diffraction (ED) [12]. In recent years, TEM resolution has been greatly enhanced to sub-angstrom level by aberration correctors [13]. Since then, TEM has provided detailed information about CNT atomic structures, such as atomic defect [14,15], chiral angle [16], atomic strain [17], interface structure [18], and handedness [19]. Complementary to the local atomic structures in real space, ED provides structural information in reciprocal space and has been used for analyzing the chirality of individual CNTs [20–25]. By analyzing layer line patterns, detailed helical structures could be understood. For example, chiral angle could be calculated from the ratio of distances of principal layer lines (D1, D2, D3) to the equator line. And by analyzing intensity oscillations in the equator line, CNT diameter could be calculated. Up to now, such microscopic analysis has been done manually, which is time-consuming and prone to human error.

In 2020, Förster et al. developed a deep learning approach for determining the chiral indices of CNTs from high-resolution transmission electron microscopy (HRTEM) images [26]. Automated assignment of the chiralities from the deep learning model was found to be in good agreement (71%) with manual analysis results. It was also noticed that the accuracy was still not sufficient, especially for nanotubes with relatively large diameters of ~2 nm.

Analytical approach, with better interpretability, has been tried to analyze the diameters from TEM images and chiral angles from fast Fourier transform (FFTs), however, with less success. It is in principle a simple process to analyze TEM images for CNTs chiralities, essentially by matching the diameter and chiral angle with a table. However, in practice, it is very challenging to accurately assign the chiral indices, mainly for two reasons. Firstly, it is still difficult to measure the diameter of CNTs from TEM images with high precision, due to the interference nature of the fringes [27,28]. Secondly, there is a compromise between resolution in real and reciprocal space. Due to the small field of view (FOV) of HRTEM images, the FFTs suffer from a noisy background.

In this work, a Python code ‘VibeTube’ is developed by using the analytical approach to extract chirality of CNTs from TEM images. Diameters are measured precisely by considering the influence of defocus and by implementing symmetry-based algorithms. Precise measurements in the reciprocal space are realized by a combination of masking, background subtraction, and iterative multiple peak fitting process. As a result, a high accuracy of 84.5% is achieved, tested against a simulated dataset, including 6930 TEM images from 330 chiralities ranging from (6, 0) to (25, 25), diameters from 0.5 nm to 3.0 nm, and defocus from −10 nm to +10 nm. In addition, by limiting the defocus value to be larger than 1 nm, to avoid the lack of contrast at near focus, the accuracy is further enhanced to 92.3%. Robustness of the code was tested against an experimental dataset with defocus ranging from −22.8 nm to +22.8 nm. With defocus limited to a reasonable range from −11.4 nm to 7.6 nm, 13 out of 14 outputs were consistent, proving the high robustness. The code has been applied to analyze the chirality distribution of CNTs grown by using a floating catalyst chemical vapor deposition (FCCVD) method with diameters ranging from ~1.4 nm to ~2.8 nm.

## Results & discussions

### ‘VibeTube’ code architecture and pipelines

The purpose of ‘VibeTube’ codes is to provide the research community with an open-source tool for accurate, robust, fast, and automated determination of carbon nanotube (CNT) chirality indices (n, m) from TEM images. It is designed to be easy to use, with the main architecture that includes interactive Jupyter notebooks and helper modules (Figure S1). In the notebooks, the user can set parameters, call helper functions, and display results. All physics and computation logics live in helper modules. Parameters are included in a metadata file, which is shared between pipeline stages, for example, between TEM simulation and chirality analysis.

The development of ‘VibeTube’ Python codes was carried out on a Windows 11 Pro system for Workstations. The hardware includes an Intel(R) Xeon(R) W-2195 CPU (2.30 GHz), 256 GB of RAM, and a graphics processing unit (GPU) of NVIDIA Quadro GV100 with a GPU memory of 32 GB. The codes were developed on Visual Studio (VS) Code with the assistance of artificial intelligence (AI) models, mainly Claude Code.

The codes have been designed for high accuracy, high robustness, and high efficiency. By analyzing the physics and contrast transfer functions (CTF) of electron microscopy, a new method is developed to measure the diameter precisely and to be robust against the change of imaging conditions, such as the defocus. Symmetry-based criterion helped to determine the fringes corresponding to the CNT walls. Quantitative measurement and fitting enable reliable diameter offset for calculating the diameter precisely. Insight into the TEM simulation process resulted in enhanced efficiency, where computation-expensive multi-slice simulation was conducted once and combined with a CTF to calculate TEM images of defocus series.

For AI-assisted coding, or ‘vibe coding’, it is important to define the architecture and conventions clearly. There are two particularly important conventions in this work: coordinates and defocus. During TEM simulations in abTEM, the coordinate mapping was as follows: nanotube axis was along x and perpendicular to y, and beam propagation was along direction z (Figure S2). In this work, defocus values were referenced to the nanotube center plane, not the cell bottom which is the native reference in abTEM. The conversion is as follows: df_abTEM = df_user + cell_width / 2, where cell_width is the supercell side length. In abTEM, positive defocus = underfocus, while in Kirkland convention: C10 = –defocus. This convention was verified by the contrast reversal (d_max/d_min crossover) occurring at df ≈ −0.5 nm for all tested nanotubes, confirming that df = 0 corresponds to Gaussian focus at the tube center.

**Figure 1.**
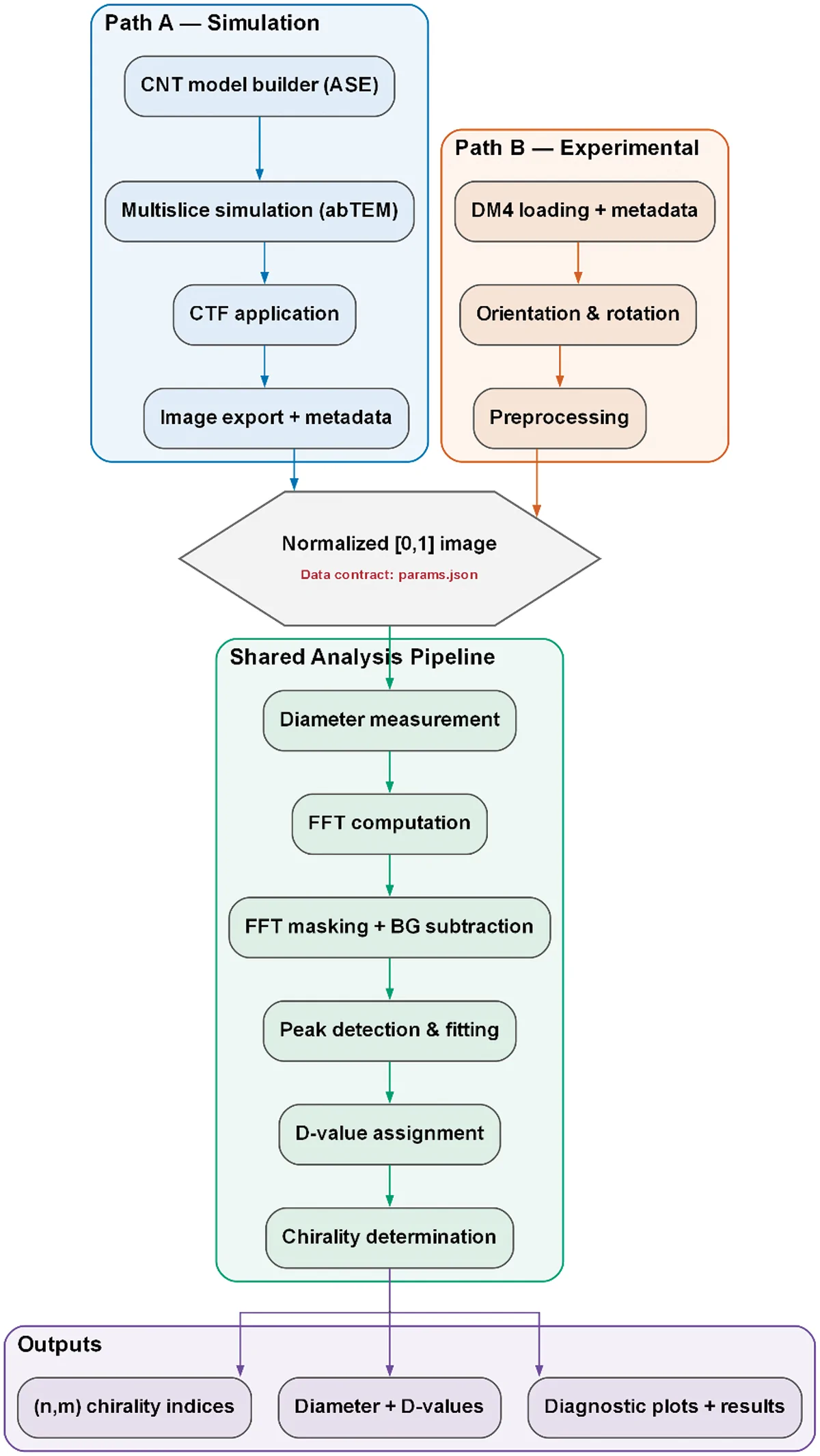
Pipelines of ‘VibeTube’ codes for extracting chirality from CNTs’ TEM images.

The basic pipeline for extracting chirality from TEM images follows (Figure 1): TEM image → diameter + FFT D-values → chirality lookup (n, m). Simulated TEM images and experimental TEM images are preprocessed into a common normalized representation, then passed through a shared analysis pipeline that extracts chirality from diameter and FFT layer line measurements. Pipeline for simulated images includes (1) multislice simulations to generate TEM images via abTEM and (2) chirality analysis to extract chirality from simulated TEM images. Pipeline for experimental TEM images includes the following: (1) preprocessing TEM images and (2) chirality analysis to extract chirality from experimental TEM images. Both pipelines share the same chirality analysis algorithms.

### Diameter measurements

To accurately assign the chiral indices of CNTs, the diameter must be precisely measured. Under weak-phase object approximation (WPOA), the TEM image of a carbon nanotube is a 2D projection of the 3D cylinder potential. At the optimum imaging condition, the focus is slightly negative to compensate for the positive spherical aberration. The focus condition is the so-called Scherzer focus. Under this condition, atoms show dark contrast in TEM images, and carbon nanotube walls are featured by two parallel dark fringes, along with bright fringes outside. Therefore, measurements of CNTs’ diameters have been done commonly by measuring the distance between the dark fringes, which is defined as d_min. However, d_min has been reported to be not precisely equal to the geometric diameter of CNTs, which is defined as d_cal in this work [26]. Förster et al. applied a diameter offset (0.06 nm) to d_min to measure the diameter of CNTs [26]. It was reported that the distance between the so-called inflection points (d_inf) between the dark and bright fringes is closer to the actual diameter; however, such inflection points are not straightforward to measure and not easily implemented experimentally.

Position of d_min is closely dependent on imaging conditions, especially the defocus value. More importantly, when the imaging condition is changed from underfocus to overfocus conditions, there is a contrast reversal. Instead of dark fringes, CNT walls will show dominant bright fringes with less dominant dark fringes outside, which is the opposite of the image taken at underfocus condition. The reason for the contrast reversal is due to the CTF, which describes the phase shift due to the objective lens: χ(k) = –π λ Δf k^2^ + (π/2) Cs λ^3^ k^4^, where λ is the electron wavelength, Δf the defocus, and Cs the spherical aberration coefficient. For an aberration corrected TEM, Δf dominates the CTF. The image intensity of a weak phase object is modulated by sin(χ), which changes sign when Δf passes through near zero (Gaussian focus), leading to the contrast reversal.

In this work, the measurement of CNTs diameters is improved by measuring d_ave, which is the average distance between the dark and bright fringes along CNT walls (d_min and d_max). While d_max and d_min swap roles at contrast reversal, d_ave = (d_max + d_min)/2 is largely immune to the swap. It was noticed that the fringes of CNT walls show symmetric patterns, that is ‘max-min-min-max’ for underfocus condition, and ‘min-max-max-min’ for overfocus condition. In addition, after subtraction of the background, the variance of the nanotube area is much higher than that of background. Therefore, an algorithm based on local variance and symmetry was designed to identify the wall fringes reliably. Then d_ave was calculated by averaging d_min and d_max, and d_inf was obtained by calculating the second derivative between each max – min pair.

**Figure 2.**
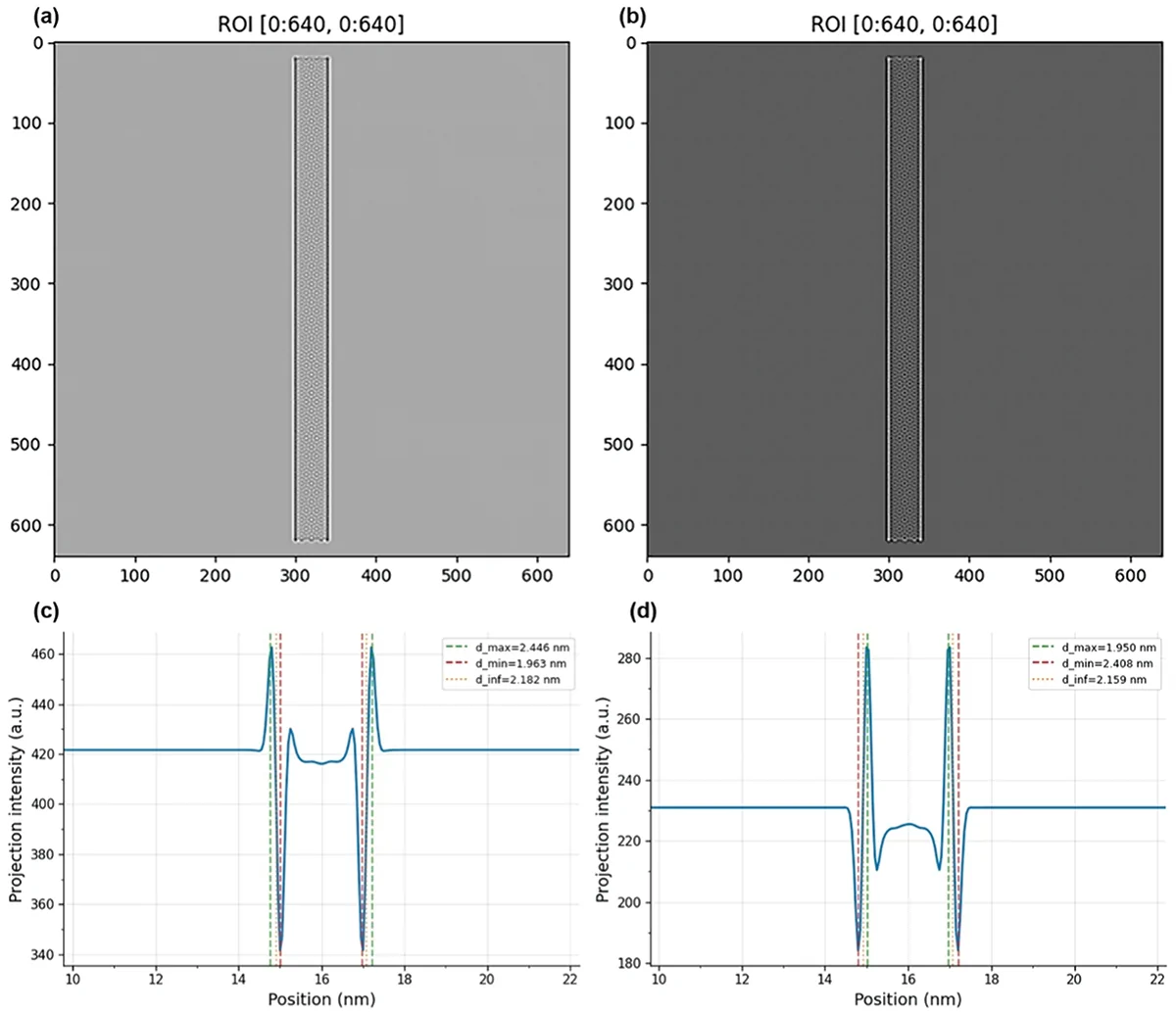
Diameter measurement by using d_ave mode. (a-b) Simulated TEM images of (20, 10) CNT at under (df = 4 nm) and over (df =  −4 nm) focus conditions. (c-d) Projected intensity profiles and detected max, min, and inflection points.

Diameter measurement results of the (20, 10) nanotube are presented in Figure 2 and Figure S3, for diameter modes of d_ave and d_min, respectively. Simulated TEM images at under- and overfocus conditions are shown in Figure 2(a,b) and Figure S3(a,b). At a defocus of 4 nm, the nanotube shows a dark fringe with a bright fringe outside. Such contrast is reversed for the image simulated at a defocus of −4 nm. Such contrast features could be clearly seen in the projected intensity profiles (Figure 2(c,d) and Figure S3(c,d)). Symmetric but opposite patterns could be detected by images at under- and overfocus conditions. For conventional d_min method, the diameter was measured to be 2.023 nm and 2.468 nm. While the measured value is close to the ground truth of 2.072 nm at underfocus condition, there was a big error (19.1%) for the d_min value at overfocus condition. On the other hand, the d_ave values were calculated to be 2.085 nm and 2.059 nm, with a small error of 0.6% and −0.6%, for under- and overfocus conditions.

Diameter modes and diameter offsets from the geometric diameters of CNTs are tested by using a simulated dataset, including 330 CNT species ranging from (6, 0) to (25, 25), and defocus values df = −10 to +10 nm, therefore 6,930 simulated TEM images. Diameter offsets, defined as the differences between measured and calculated diameters, were evaluated for both under- and overfocus conditions to determine the most stable, reliable, and implementable diameter mode. Optimum diameter offsets for each diameter mode have been determined from the average offsets.

**Figure 3.**
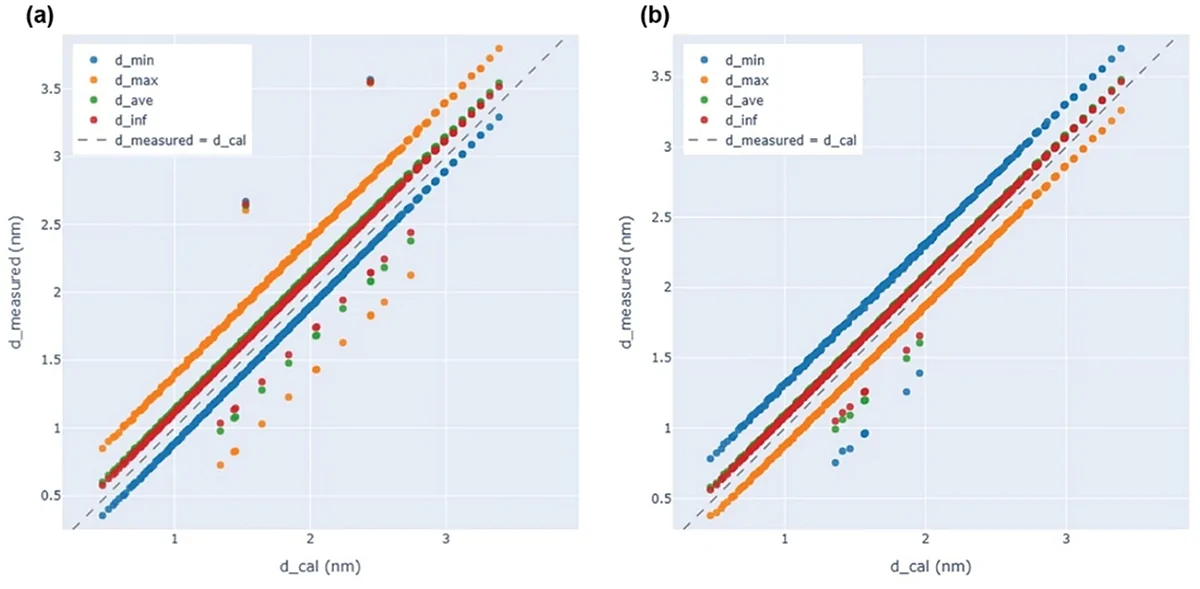
Diameter-offsets for different measurement methods. (a) Measured d_min, d_max, d_ave, and d_inf as functions of d_cal with defocus of 2 nm (underfocus). (b) Measured d_min, d_max, d_ave, and d_inf as functions of d_cal with defocus of −2 nm (overfocus).

Figure 3 presents the diameters determined by different modes against the geometric diameters of the simulated dataset at under (a) and over (b) focus conditions, for defocus values of 2 nm and −2 nm, respectively. Except for a few points, most of the points showed linear relations with the actual nanotube diameters, for both under- and overfocus conditions. It is interesting to note that for d_min (blue) and d_max (orange), there was a swap between them for the two focus conditions. Such swap can be understood by contrast reversal due to the change in sign of phase shift in the CTF. On the other hand, d_ave and d_inf are almost overlapped, and the offsets of these two modes did not change the sign along with the change of focus. Therefore, for the conventional d_min mode, it is only reliable for the underfocus condition, with an offset of 0.10 nm. In this work, we use d_ave to measure CNT diameters, with an offset of −0.12 nm. This optimum diameter offset was found largely independent of defocus condition for a [−10, +10] nm defocus range.

### FFT measurements

In addition to diameter measurements in real space (TEM images), the chiral angles are calculated based on layer line measurements in reciprocal space, which are the Fourier transform of TEM images. Because of the small area of a TEM image, the FFT is typically noisy, making it difficult to precisely measure the featured lines corresponding to CNT diffractions. In conventional FFTs, periodic boundary conditions are assumed for the systems, which is not the case for the TEM image. As a result, artifacts due to the edges obscure the weak layer line from CNTs. We have used Moisan’s periodic + smooth decomposition method to eliminate the edge artifacts [29]. A 2D polynomial background is fitted to the masked square region (±6.5 nm^−1^) and subtracted to flatten the residual gradient. Clear layer lines could be seen from processed FFTs. As expected, FFTs are not sensitive to the focus conditions (Figures S4-S5).

The distances of layer lines (D1, D2, D3) to the equator were measured by fitting the projected intensity profile with multi-peak pseudo-Voigt model plus a linear baseline. D1/D2 and D3 regions were separately fitted in an iterative algorithm up to six iterations. One of the difficulties is the small separation between D1 and D2 for near armchair type region. Peaks with fitted width larger than 3 px were split into two narrower peaks, to resolve close peaks near armchair chiralities.

D1 and D2 layer lines were tentatively assigned to two strong peaks within D1/D2 range [2.20, 4.84] nm^−1^. Then, the assignment was validated by calculating the chiral angle α = arctan ((2D2 − D1) / (√3 D1)) to check if α lies in [0°, 30°]. Another cross-check was to compare D3 = D1 − D2 with measured D3 peak and flag the consistency between the two. Finally, special case of armchair (D1 ≈ D2) was considered if there is no D3 peak detected corresponding to the D1-D2, and if there is only a dominant peak in D1/D2 range. Chiral angles of the CNTs are calculated by using the layer line method [24].

**Figure 4.**
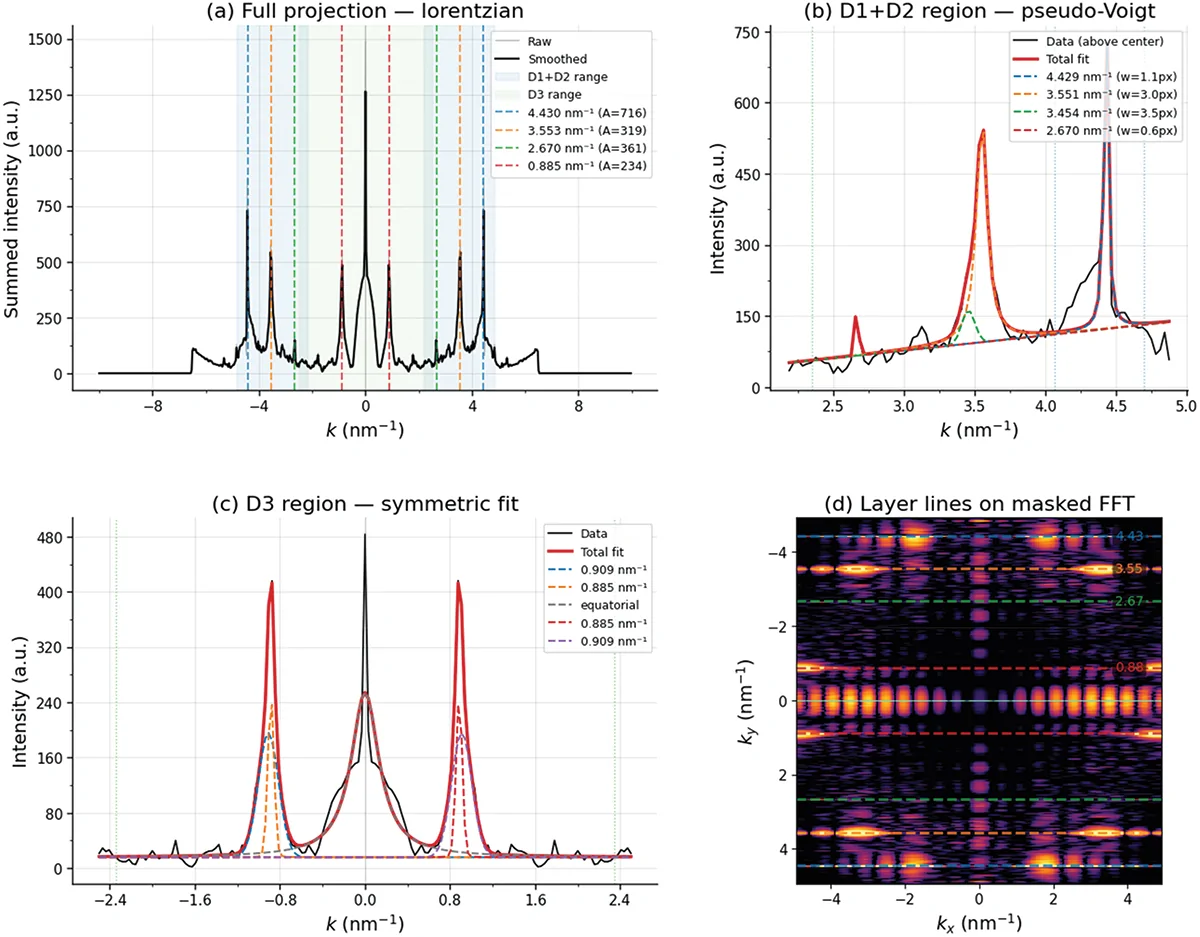
Layer line detection and measurement of the FFT from (20, 10) CNT at an underfocus condition. (a) Projection profile of the processed FFT with multiple peaks detected. (b) Fitting and peak detection in D1/D2 region by using a pseudo‑Voigt method. (c) Fitting in D3 region by using the symmetric algorithm. (d) FFT in the central area with detected peak positions marked, corresponding to the peaks in (a).

Example of the layer line measurement is presented in Figure 4 and Figure S6 for the simulated TEM image of (20,10) nanotube. In addition to the equator line, four peaks were detected on either side of the projected profile of the processed FFT (a), as marked by the dotted lines. The profile was further fitted in D1/D2 and D3 regions, where it is expected to find two peaks in D1/D2 region for calculating the chiral angle and to find a peak in D3 region to validate the two peaks in D1/D2 region. The FFT in the central area, along with detected layer line positions, corresponding to the peaks in Figure 4(a) is plotted in Figure 4(d). Among the four detected peaks, three of them were identified as D1, D2, and D3 to satisfy the D1-D2 = D3 requirement.

### Chirality analysis of simulated TEM set

Based on precise measurement of diameter, layer lines, and subsequently chiral angles, chiral indices of the CNTs were determined. Experimental circumference was calculated by Ch_exp = diameter_nm × π, and experimental chiral angle was calculated using α_exp = arctan ((2D2 − D1) / (√3 D1)). Then, the experimental circumference and chiral angle were compared with pre-built lookup table, to find the best matched indices that satisfied with the following conditions: |Ch_calc − Ch_exp| / Ch_exp < 7% AND |α_calc − α_exp| <2.0°. The tolerance of acceptable circumference and angles can be adjusted in the analysis Jupyter notebooks, depending on specific calibration of the electron microscopes.

An example of chirality analysis of a simulated TEM image from CNT (20, 10) model is presented in Table 1. Analysis results from conventional d_min and the measurement mode used in this work (d_ave) are compared for under- and overfocus conditions. As the defocus value was varied from −4 nm to 4 nm, the measured chiral angle was very robust, showing a small deviation of 0.02 degrees and very close to the actual angle of 19.1 degrees. On the other hand, the measured diameters were dependent on measurement modes and defocus values. For conventional d_min mode, while the measured diameter was close to the ground truth at underfocus condition, it showed a large error for the overfocus condition. On the other hand, d_ave values were robust against the change in defocus. As a result, the chiral indices were correctly assigned for d_ave mode at both focus conditions, while the result for d_min mode was only correct at underfocus condition.

**Table 1.** Chirality analysis of a simulated TEM from CNT (20, 10).

CNT model	Defocus (nm)	Diameter mode	Diameter (nm)	Alpha (deg)	n	m	Match
(20, 10)	4	d_ave	2.08	19.22	20	10	1
(20, 10)	−4	d_ave	2.06	19.24	20	10	1
(20, 10)	4	d_min	2.06	19.22	20	10	1
(20, 10)	−4	d_min	2.51	19.24	24	12	0

**Figure 5.**
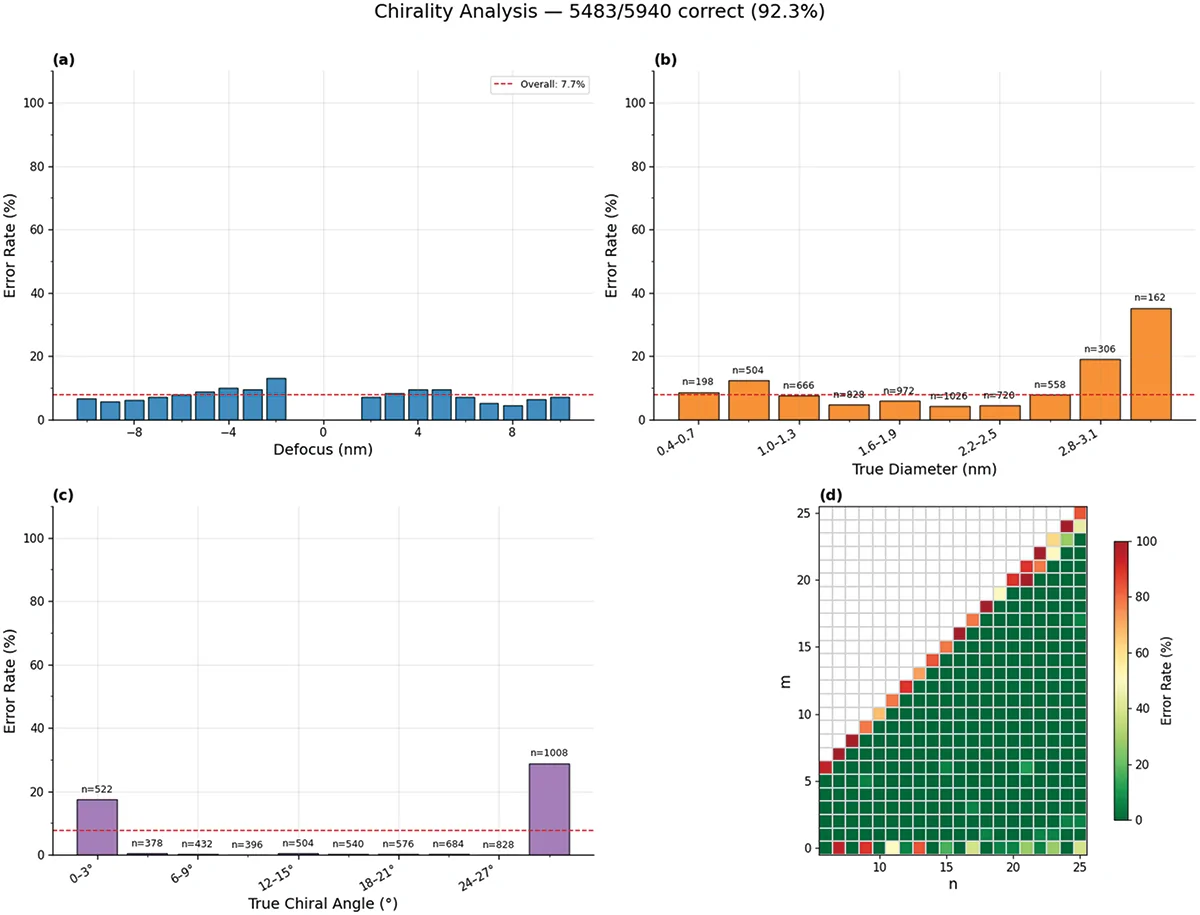
Error analysis for simulated dataset with diameter measured by using d_ave mode and excluding |df| < 2 nm. (a) Error rate as a function of defocus. (b) Error rate as a function of tube diameter. (c) Error rate as a function of chiral angle. (d) Error rate as a function of chiral indices (n, m).

Accuracy, robustness and efficiency of the ‘VibeTube’ code have been tested using the simulated dataset, including 330 chiralities, ranging from (6, 0) to (25, 25), and 6930 simulated images in total. Statistical analysis results are presented in Figure 5, and Figure S7-S9, for different diameter modes and defocus ranges. For d_ave diameter mode, the overall accuracy was 84.5% (Figure S7), which was significantly higher than the accuracy of 44.9% by using the conventional d_min mode (Figure S8). It is interesting to notice that the accuracy of d_min mode was very high (>90%) in underfocus conditions; however, the error rate was nearly 100% for overfocus condition, due to the error in diameter measurement.

For d_ave mode used in the current work, the error rate was also very high when the defocus is near zero. It is understandable that when the focus is close to the Gaussian point, the contrast is minimized, causing confusion in diameter measurement. Therefore, it is recommended to avoid the near focus condition to take TEM images of CNTs. Within the range 2 nm < |df| < 10 nm, the accuracy was as high as 92.3% (Figure 5). The accuracy was rather robust against the change in defocus (Figure 5(a)), in contrast to the behavior of d_min mode (Figure S9). As the diameter increases, it becomes more difficult to differentiate neighboring indices, and the error rate increases for nanotubes near 3 nm in diameter (Figure 5(b)). Nanotubes near zigzag and armchair regions are more challenging than chiral nanotubes (Figure 5(c)), mainly because of the special diffraction patterns with layer lines that are difficult to separate. Such chiral angle-dependent error rate was also clear from the chiral map (Figure 5(d)).

Within the recommended window (2 < |df| < 10 nm, accuracy 92.3%), the residual error is structural and concentrates on near-armchair and near-zigzag tubes (Figure 5(c,d)). The chiral angle is obtained from the positions of the D1, D2, and D3 layer lines (D3 = D1 − D2). And these lines coalesce at the two limits of the chiral-angle range – D1 and D2 merge toward armchair (α → 30°, D3 → 0), and D2 and D3 merge toward zigzag (α → 0°, D1 → 2·D2). When the layer lines get too close, they cannot be resolved reliably. The diameter, by contrast, is measured within tolerance. So, it is the chiral angle measurement, not the diameter, that limits accuracy in this regime. The pipeline handles these special cases with symmetry constraints, a D1 − D2 = D3 consistency check, and a dedicated armchair branch and flags ambiguous cases for user inspection; a fully automatic treatment of the armchair/zigzag limit is left to future work.

### Chirality analysis of experimental TEM set

**Figure 6.**
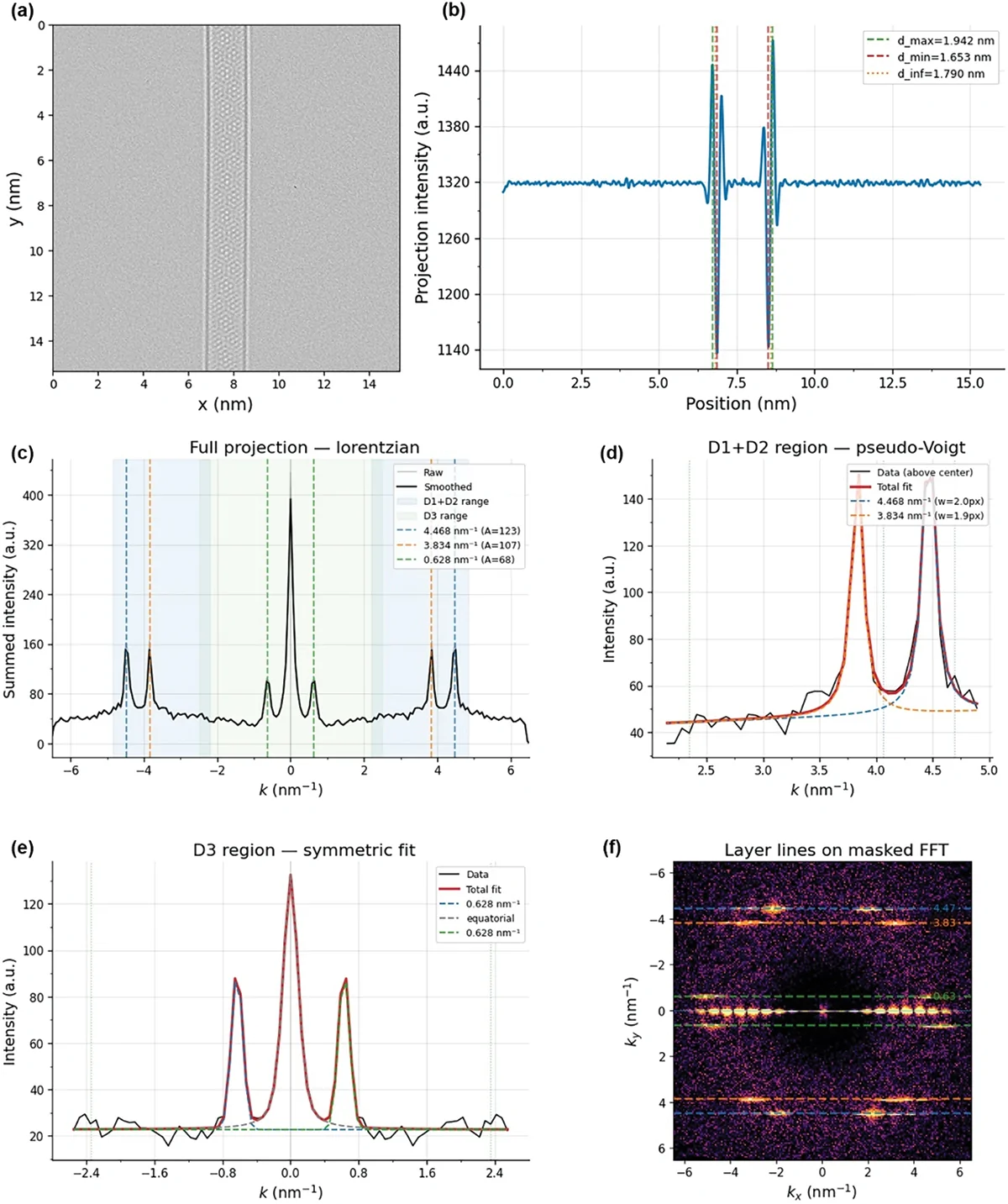
Chirality analysis of an experimental TEM image of CNT. (a) Preprocessed TEM image. (b) Projected profile and detected d_min, d_max, and d_inf for evaluating the diameter. (c) Projected profile of FFT layer lines and detected peaks. (d-e) Peak fitting and detection of D1, D2, and D3 layer lines. (f) Processed FFT with detected D1, D2, and D3 layer lines labelled.

Accuracy and robustness of the ‘VibeTube’ code were further tested on an experimental dataset of HRTEM images of CNTs synthesized by an FCCVD method. Figure 6 shows the step-by-step treatment and analysis of one TEM image example after preprocessing. The background corrected image in Figure 6(a) shows extremely clear CNT features on a very homogeneous background. Normalized image intensity is then projected along the tube axis and used for the walls detection, as shown in Figure 6(b). Several oscillations corresponding to the two wall regions are clearly visible above a flat background. Those oscillations are used to determine d_max, d_min, and d_inf. While in this example the detected peaks correspond to the global maxima, the peaks are not simply assigned by using the maximal oscillation variation on each side. All fringe peaks above a prominence threshold are considered, and the pair that best matches the expected wall symmetry (max – min – min – max at underfocus, reversed at overfocus) is selected. Following their detection, the average between d_max and d_min was calculated for d_ave. Following the CNT diameter extraction, boundary corrected and center-masked FFT of the preprocessed image was generated (Figure 6(f)) and its intensity projected along the equatorial line (Figure 6(c)). The signal is then smoothed and fitted to detect peaks in three distinct ranges of the projection. The first two ranges correspond to the two pairs of so-called D1 and D2 lines, on both sides of the equatorial line. Figure 6(d) shows an enlarged view of one range and the fits corresponding to D1 and D2. The third range is centered on the equator and includes the D3 lines pair, as enlarged in Figure 6(e). Symmetry conditions on the pair peaks are applied here as well to account for the physics of electron diffraction of tubes. As visible in Figure 6(c,f), the lines reported on the FFT and its projection, allowing users visual validation, are properly assigned. The chiral angle α is then calculated from the D1 and D2 line positions. Finally, the as mentioned determined diameter and angle couple is compared to the theoretical couple list for CNTs, and the chirality extracted from the best matching couple. In this example, a d_ave of 1.678 nm has been detected, and D1 and D2 positions have been determined at 4.468 nm^−1^ and 3.834 nm^−1^ giving α = 22.5°, best matching a (15, 9) chirality.

#### Robustness: defocus

**Figure 7.**
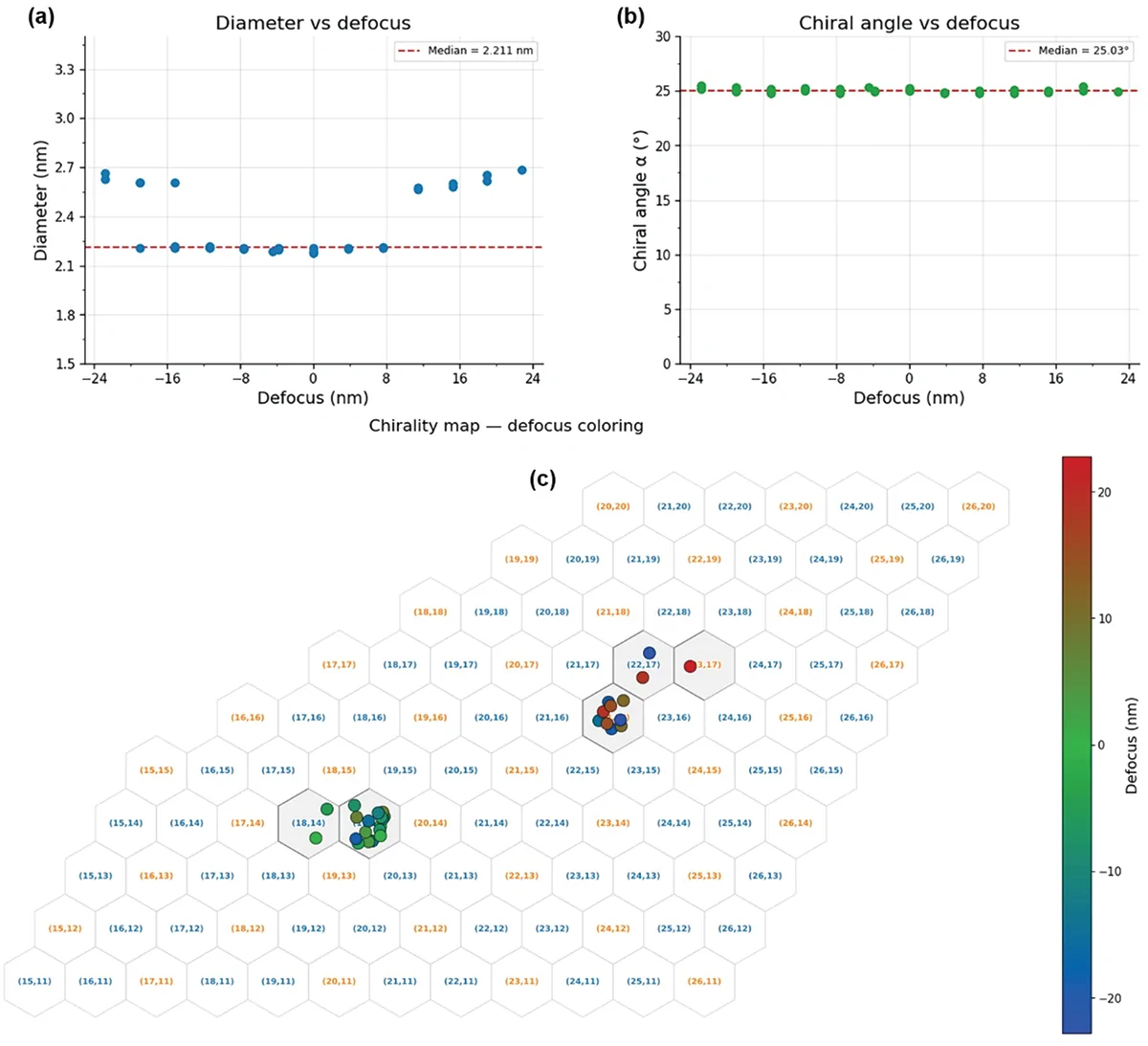
Robustness test of ‘VibeTube’ codes on a CNT (experimental image) with defocus series. (a) Measured diameter as a function of defocus. (b) Measured angle as a function of defocus. (c) Calculated (n, m) as a function of the defocus, where the defocus value is indicated by a color bar.

Robustness against defocus of the ‘VibeTube’ code was tested with 31 images of one CNT, recorded at 14 different focus conditions, with defocus value ranging from −22.8 nm to +22.8 nm. The measured diameters are about ~2.2 nm for the defocus in the range from −11.4 nm to 7.6 nm. When the defocus was out of the range, the measured diameters showed a jump to ~2.6 nm (Figure 7(a)). It is understandable that as the focus is increased either in the under- or overfocus directions, more fringes will appear, making the identification of the correct wall positions difficult. Chiral angles are distributed within a narrow range of 25.1 ± 0.4°, showing a higher robustness against the focus conditions (Figure 7(b)). Extracted (n, m) indices are presented in Figure 7(c). Since diameter measurements are correct only within the defocus ranging from −11.4 nm to 7.6 nm, it is not surprising that the chiral indices also showed discontinuity at these defocus values. Within the defocus range [−11.4, 7.6] nm, 13 out of 15 images were assigned to (19, 14) and 2 were assigned to (18, 14). Since one of the (18, 14) results from the image with defocus at 0 nm, at which point the contrast is very low, the accuracy of the code in the proper defocus range is estimated to be 13/14 = 92.9%, which is consistent with the estimated accuracy from simulated dataset.

#### Statistical analysis and chirality map

**Figure 8.**
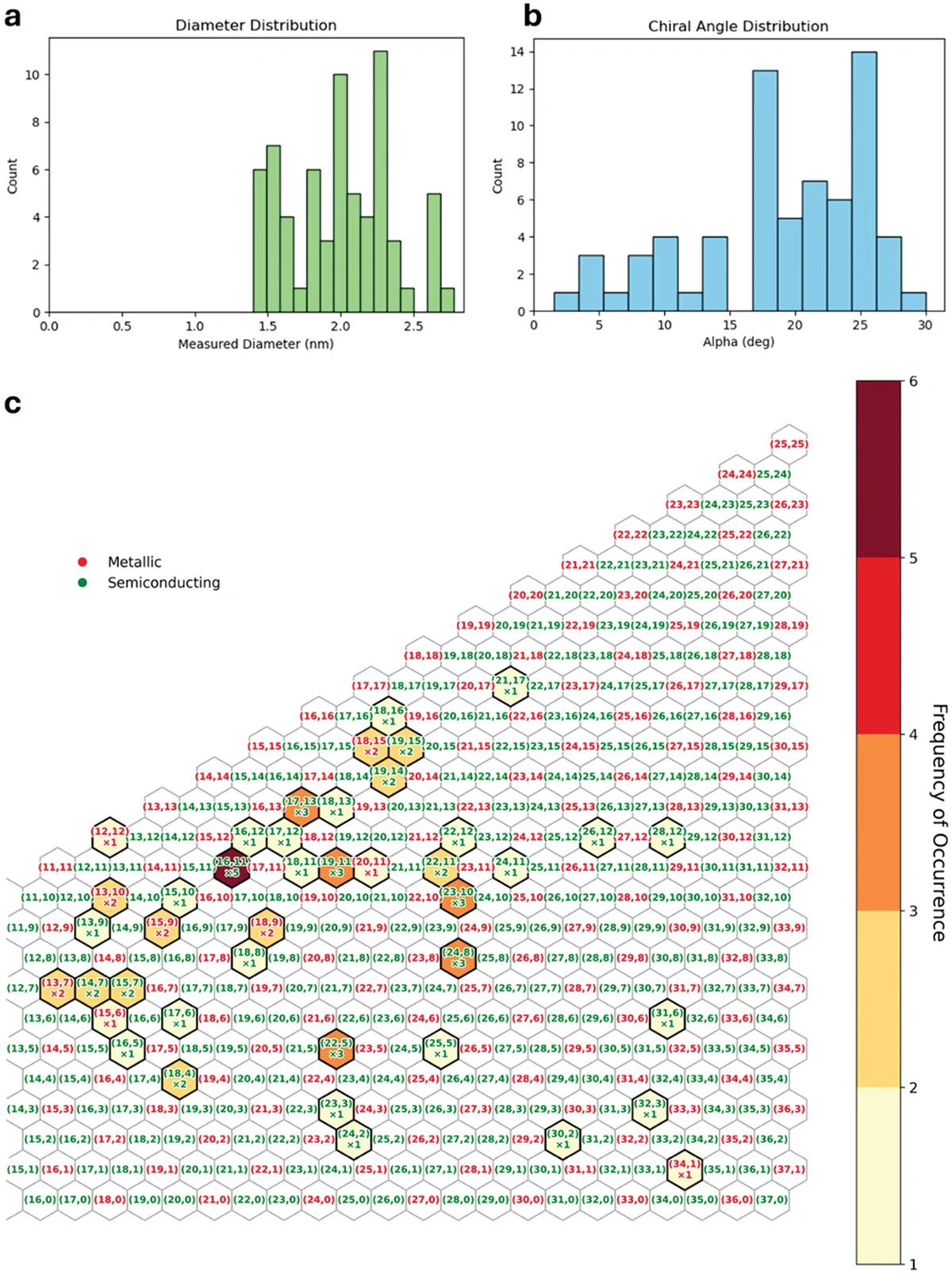
Statistical analysis of chirality distribution of CNTs grown by FCCVD. (a-b) Distribution of measured diameters and chiral angles. (c) Chirality distribution with the frequency of occurrence indicated by the color.

The ‘VibeTube’ code was used to analyze the chirality distribution of CNTs grown from FCCVD method [30], with a dataset of 67 TEM images from 50 unique CNTs. Images have been selected based on their quality, so that clear FFT patterns can be seen. As can be seen in Figure 8, the code outputs a distribution of 42 chiralities, with diameters ranging from ~1.4 nm to ~2.8 nm and chiral angles spread from ~1.6° to 30°. For 17 of the CNTs, two different images of the same tube have been included to test the consistency. In all 17 cases, extracted chiralities are consistent for the two input images, showing the high reliability for practical applications.

### Extension to double-walled CNTs and bundles

The pipeline of chirality extraction from TEM images is designed and validated for isolated single-walled carbon nanotubes. It detects the two wall fringes that define one diameter and the three layer lines on each side of the FFT equator that define one chiral angle. In practice, there are double-walled CNTs, and SWCNTs typically exist in the form of bundles. Accurate chirality assignment of more complex CNT materials is the basis to investigate the rich physics of the interactions and to realize their chirality-dependent applications. Previously, electron diffraction has been applied to extract the chirality of each shell in multi-walled CNTs [22,31,32]. Here, we have explored an extension of the method from isolated single-wall CNTs to extract the chirality of double-walled CNTs and bundles from simulated TEM images.

Although the analytical pipeline developed based on isolated SWCNTs does not apply directly to cases with more than one nanotube shell such as DWCNTs and bundles, the underlying measurements can be extended naturally. In the TEM image of a multi-wall CNT or multi-nanotubes, the number of wall fringes and layer lines in FFTs is proportional to the number of nanotube shells. One of the difficulties is detecting and pairing the wall fringes in TEM images with the layer lines in FFTs. It is noticed that the larger diameter shell contains more atoms per projected length and therefore produces both the deeper wall fringes in TEM images and the higher intensity layer lines in FFTs, so a larger diameter can be paired with the stronger layer line set to determine the chirality of each shell. The above process was implemented as an additive module, leaving the validated single-wall pipeline unchanged. The analysis was tested on frozen-phonon multislice TEM simulations of a (10,5)@(20,3) DWCNT and a (10,5)+(20,3) bundle under different overlapping conditions.

Chirality indices of the nanotube shells in the DWCNT and bundle were extracted (Figures S10 and S11). For the DWCNT, the four dark wall fringes were detected in the projected wall profile and grouped by contrast depth. At underfocus condition, the diameters were measured by using the dark-fringe d_min mode, which avoids the ambiguous bright/dark pairing when several walls overlap. This gives two correct diameters (1.73 nm and 1.07 nm), without prior knowledge of how the walls are arranged in projection. The FFT shows six layer lines (two in each of the D1, D2, and D3 sets). Taking the two strongest peaks per set and pairing them by the same D3 = D1 − D2 consistency yields two (D1, D2) triplets and hence two chiral angles (6.9° and 19.3°). The larger measured diameter was paired with the stronger layer line triplet. The result was (20,3) for the outer shell and (10,5) for the inner shell, matching the model structure exactly.

For the bundle, the chirality was extracted through a similar procedure. The chiral angles were robust against the overlapping conditions, because the FFTs are essentially identical irrespective of projected overlap, with the layer lines of both tubes superimposed. The diameters were measured correctly for the full-overlap and no-overlap cases, giving (20,3) and (10,5) for the two nanotubes in the bundle. Partial overlap was the most difficult case: the wall fringes interfere in the overlapped region, so the measured diameters were slightly off (1.74 nm and 1.08 nm). The larger tube was then assigned (21,3) rather than (20,3), one index off, while the smaller tube’s chirality remained correct. These results show that, in principle, the pipeline designed for isolated SWCNTs can be extended to more complex CNT materials such as double-walled tubes and bundles. However, further work, using both simulations and experiments, is needed to address the wider range of conditions encountered in practice.

### Practical usage guideline

For practical applications of the VibeTube codes to analyze TEM images to extract CNTs’ chiralities, it is important to understand how the imaging parameters affect the data quality and accuracy. Practical usage guidelines have been made based on the investigations of the influence of three parameters, electron dose, pixel size, and field of view (Figure S12), in addition to defocus influence, which has been discussed in previous sections.

The robustness test was based on simulation [33] and analysis of a dataset with nanotube indices ranging from (20, 1) to (20, 19), spanning from near-zigzag to near-armchair ranges. The electron dose was applied as Poisson shot noise on the intensity. The pixel size was coarsened by area-averaged down-sampling. The field of view was reduced by re-simulating the tube at each length. The full procedure and code are provided with the released analysis notebook.

Electron dose (Fig. S12a) sets the overall signal-to-noise: the assignment is fully correct for doses ≥ 1000 e/Å^2^ – the range of standard HRTEM imaging (500–2000 e/Å^2^, shaded) – and degrades below a few hundred e/Å^2^, where the weak layer lines are the first features to disappear under the shot noise background. Pixel size (Fig. S12b) limits the diameter measuring precision: as the sampling coarsens, the wall fringes are blurred, and the diameter is overestimated. The accuracy stays ~95% up to 0.05 nm/px and collapses beyond 0.06 nm/px. Tube length (Fig. S12c) limits the layer line measuring precision: a shorter tube has fewer lattice periods, so the layer lines broaden. The accuracy is 100% for lengths ≥15 nm but falls sharply for shorter tubes. The two-parameter maps (Fig. S12d,e) show that a high accuracy is associated with a high dose ≥ ~1000 e/Å^2^, a small pixel size ≤0.05 nm/px, and a long nanotube ≥15 nm. Together with defocus 2 < |df| < 10 nm, these parameters are the recommended conditions for accurate analysis of CNTs’ chirality from TEM images.

## Conclusions

In conclusion, an open-source Python pipeline is developed for automated chirality extraction of carbon nanotubes from TEM images with an accuracy exceeding 90% across diameters from 0.5 to 3.0 nm against a simulated dataset. The method combines symmetry-based diameter measurement with sub-pixel layer line analysis in Fourier space, enabling reliable extraction of chiral indices (n, m). Validation with CNTs synthesized by floating catalyst CVD confirms the practical applicability of the codes to real experimental datasets. By releasing the ‘VibeTube’ code as an open-source tool, we provide the CNT research community with an accurate, robust, and efficient method for chirality characterization that can accelerate chirality-controlled growth and structure – property relationships.

## Supplementary Material

Supplemental Material

## Data Availability

The data that support the findings of this study are openly available in NIMS-operated Materials Data Repository (MDR).

## Code deposition

The “VibeTube” code is open source and available on GitHub at https://github.com/tangdaiming/VibeTube-0 under the MIT License. The repository contains the full analysis and simulation pipeline together with the notebooks that reproduce the analyses reported in this paper, including the robustness study and the DWCNT/bundle extension.
