# Supplementary material for: VibeTube: a code for accurate extraction of CNTs’ chirality from TEM images
Source: Sci Technol Adv Mater. 2026 Jul 20;27(1):2705816. doi: 10.1080/14686996.2026.2705816 (PMC13495031; doi:10.1080/14686996.2026.2705816)
Supplement: Supplementary file 1 [file TSTA_A_2705816_SM2248.docx]

# Supporting information

## Methods

### 1. SWCNTs growth and collection

SWCNT networks were prepared by an injection floating catalyst chemical vapor deposition (FCCVD) method and transferred onto TEM grids directly. The detailed synthesis process can be found in previous report (*32*). Briefly, CVD growth was conducted inside a horizontal tubular furnace. C_2_H_4_ and H_2_ were used as the carbon source and carrier gas, respectively. Floating catalyst precursor (ferrocene) was injected into the reactor by syringe pump. Temperatures in the furnace and at the position of the injection needle were 1100 °C and 83 °C, respectively. Collection time was about 20 seconds to obtain SWCNT networks containing high-purity, individual nanotubes.

### 2. Preprocessing TEM images

The TEM used in this work was a JEOL JEM-ARM300F operated at 80 kV, with aberration correctors for both TEM and STEM modes. TEM images were taken using a Gatan OneView camera, with 4k x 4k resolution. To take lattice-resolved TEM images of carbon nanotubes, the magnifications were set to 1 million to 2 million times. To obtain TEM image of defocus series, the focus was changed by objective lens focus.

Experimental TEM images of CNTs were preprocessed as a separate step, to produce the same normalized format as simulated images, and to prepare an experimental dataset that can be reused for various analysis. Through preprocessing experimental and simulated TEM images can use identical analysis pipeline to extract CNT chirality.

The pipeline for preprocessing mainly includes (1) tube orientation in coarse and fine modes, (2) rotation to vertical orientation, (3) Region of Interest (ROI) selection, (4) Background subtraction and normalization. A two-stage orientation detection was used to precisely rotate the TEM image so that the nanotube axis is along the vertical direction. In stage 1 (coarse): the user clicks two points along the tube axis in a matplotlib GUI window. The orientation angle is computed as atan2(dy, dx) from the clicked points. In stage 2 (fine sweep): an area defined by the user is rotated within 5° around the coarse angle in 0.1° steps. At each step the projection standard deviation was computed. The orientation angle was determined at the angle with maximum standard deviation in the projection profile. As a result, the orientation was accurate to ~0.1°. Two measures have been taken to improve robustness: (1) a margin of max(H,W)×sin(θ)/2 pixels was cropped to eliminate edge artifacts; (2) Gaussian background subtraction (σ=10) is applied to remove gradients in background that would otherwise dominate the signal.

### 3. Simulation of TEM images

High-resolution transmission electron microscopy (HRTEM) images of single-walled carbon nanotubes (SWCNTs) were simulated using the multislice method. The simulated images were used as a dataset for testing diameter measurement methods and for calibrating the diameter offset during chirality analysis. The dataset was used for testing the accuracy, speed and robustness of chirality analysis codes.

Nanotube models were constructed by using Atomic Simulation Environment (ASE), from chirality indices (n, m) with C–C bond length 1.42 Å. The original ASE's ase.build.nanotube() function has a hard-coded limit *nk* = 6000 that prevents construction of large-index tubes. A runtime patch was made in this project to avoid the *nk* limit, so that all (n, m) nanotube models could be constructed. The size of the cube-shaped supercell was 32 nm. The real space sample was 0.5 Å/px. And the lengths of nanotubes were 30 nm for all chiral indices.

The chiral indices ranged from (6, 0) to (25, 25), with a diameter ranging from 0.47 nm to 3.39 nm, corresponding to 330 unique chiralities. For each nanotube, the defocus varied from -10 to +10 nm. Therefore, 21 TEM images were simulated for each nanotube, and the dataset included 6,930 simulated TEM. An incremental save mechanism was used to write the summary.csv every 50 tubes to avoid data loss in case of crash.

The simulation uses the frozen phonon, multislice algorithm implemented in abTEM (*33*). Multiple atomic configurations with random thermal displacements (RMS σ = 0.1 Å) were generated to simulate thermal diffuse scattering. A plane electron wave at the specified energy (60 kV) is propagated slice-by-slice, alternating transmission (phase grating) and free-space propagation (Fresnel propagator via FFT) to obtain a complex wave function at the exit surface. TEM images were generated by converting the exit wave through the contrast transfer function (CTF): I(r) = |FT⁻¹[ FT[ψ_exit] × CTF(k)]|², where mainly the influences of defocus (Δf) and spherical aberration (Cs, 1 μm) were included.

It is noticed that the exit wave does not depend on the CTF. For a defocus series of N values on the same (n, m) tube, the exit wave was simulated once and then applied N times with the CTF including a defocus value. The defocus convention in this project is based on the reference of the nanotube center, instead of the bottom surface of the supercell, which is the convention of abTEM software. Therefore, corresponding to the defocus ranging from -10 nm to 10 nm, the CTF defocus at cell bottom was from +6.0 nm to +26.0 nm.

Since there are multiple Fourier transformations, GPU has advantage over CPU for TEM image simulations. On the specific hardware platform, the GPU was tested to be 14.5 times faster than the CPU with 1 core, and about 4.5 times faster than the CPU with 16 cores. For the simulations carried out by CPU, the performance plateaued at 16 workers due to saturated memory bandwidth. The time to simulate the 21 TEM images for one nanotube was about 7.8 seconds. And the total time to simulate the 6930 TEM images for the 330 nanotubes was about 43 minutes.

### 4. Measurement of CNT diameter

Precise measurement of CNT diameter is basic but practically complex and challenging. In this work, different approaches have been tested to get accurate and robust diameter measurements. The four diameter modes are: (1) d_max: distance between two intensity maxima, corresponding to the bright-fringe spacing, (2) d_min: distance between two intensity minima, corresponding to the dark-wall spacing, (3) d_inf: distance between inflection points, corresponding to the steepest-gradient positions, and (4) d_ave: (d_max + d_min) / 2, the average value of d_max and d_min.

The most challenging task for diameter measurement is the detection of the correct fringes that are associated with CNT walls. With the focus condition to be under focus (df>0) it is expected that the CNT wall is dominated with a dark fringe along with a weaker bright fringe outside. The contrast is reversed when the focus is changed to be over focus condition. What makes it more complex is that between CNT walls, there are also interference fringes due to the lattices of upper and lower walls. Such fringes may show strong contrast, and sometimes even stronger than the wall fringes. Therefore, simple algorithms based on global detection of intensity maxima do not work.

In this work, a new algorithm was designed to detect the correct wall fringes by using symmetric patterns and local variance. The pipeline for diameter measurement mainly includes: (1) Smooth projection with Gaussian filter (σ = 2 px), (2) Estimate wall regions from local variance, (3) Dual search for wall fringes: Path A anchors on wall minima and finds symmetric maxima; Path B anchors on wall maxima and finds symmetric minima — the path with the higher symmetry score wins; (4) Sub-pixel refinement by a parabola fitting; (5) d_min, d_max and d_ave calculation with detected maxima fringes; (6) d_inf was obtained by finding the zero-crossing of second derivative between max–min pair.

### 5. Detection of FFT layer-lines and chirality assignment

The chiral angle is calculated in reciprocal space from the layer lines of the fast Fourier transform (FFT) of the TEM image. The pipeline for FFT layer-line detection mainly includes: (1) a boundary-corrected FFT computed with Moisan's periodic-plus-smooth decomposition to remove the edge-artifact cross; (2) refinement of the equator to the brightest row within ±2 px of the geometric center; (3) masking to a square of half-width 6.5 nm⁻¹ about the center, followed by a two-dimensional polynomial background fit and subtraction to flatten the residual gradient; (4) projection of the processed FFT onto the layer-line axis (perpendicular to the equator) to give a one-dimensional profile; (5) fitting of the profile with a sum of pseudo-Voigt peaks plus a linear baseline, with the D1/D2 and D3 regions fitted separately and iteratively (up to 6 iterations, initial peak width 2 px). Detected peaks are kept only within [0.5, 4.85] nm⁻¹, with width 0.3–5.0 px and amplitude above 5 % of the profile maximum; any peak wider than 3 px is split into two narrower peaks so that close near-armchair D1/D2 lines can be resolved.

The layer lines are then assigned as follows: (1) peaks within the combined D1/D2 range [2.20, 4.84] nm⁻¹ are taken as layer-line candidates; (2) the two strongest by intensity are selected, and the higher-frequency one is assigned D1, the lower D2 (D1 ≥ D2 by definition); (3) the assignment is validated by requiring α = arctan[(2D2 − D1)/(√3 D1)] to lie in [0°, 30°]; (4) D3 is cross-checked in the range up to 2.35 nm⁻¹: a measured D3 peak that matches D1 − D2 within 2 Δk confirms the (D1, D2, D3) consistency; (5) special cases near armchair region are handled explicitly — if D1 ≈ D2, or if a single dominant peak is found in the D1/D2 range with no real peak in the D3 region, the tube is flagged as armchair type (D2 = D1, D3 = 0); (6) the chiral angle is computed from the validated D1 and D2, and the (n, m) indices are assigned from the diameter and chiral angle.

### 6. Simulation and analysis of DWCNT and CNT bundle

Multi-tube models were built from the same fixed-geometry single-wall builder (ASE, with C–C bond length 1.42 Å). The DWCNT was constructed as two concentric shells, inner (10,5) and outer (20,3), The bundle was constructed from tubes (10,5) and (20,3) placed parallel and kept in contact at a fixed centre-to-centre distance of 1.706 nm. The projected (in-image) separation Δy was set to 0, 0.85 and 1.60 nm to give full, partial and no projected overlap.

TEM images were simulated with the frozen-phonon multislice method with 8 configurations, RMS displacement σ = 0.1 Å, plane-wave illumination, exit wave computed then combined with the contrast transfer function. The accelerating voltage was 60 kV, and the pixel size was 0.0127 nm/px, fine enough to resolve the ~0.33 nm inter-wall spacing. Images were simulated at +4 nm defocus (underfocus).

The projected wall profile was obtained. A DWCNT or two-tube bundle presents four dark wall fringes rather than two. These were detected and grouped by contrast depth, the two deepest fringes belonging to the larger-diameter shell and the two shallowest to the smaller. Each shell diameter was taken as the separation of its two dark fringes (d_min mode) plus a fixed 0.10 nm offset, which is accurate at underfocus condition.

The FFT was computed and cleaned as for a single tube following periodic-plus-smooth decomposition, central-area masking, and polynomial background subtraction. A two-shell nanotube (either DWCNT or bundle) produces six layer-lines, two in each of the D1, D2 and D3 sets. In each set the two strongest peaks were retained, and the six peaks were paired into two (D1, D2, D3) triplets using the same D3 = D1 − D2 consistency as the single-wall method. Each triplet gives one chiral angle through the standard relation α = arctan[(2D2 − D1)/(√3 D1)].

The two diameters and two chiral angles were paired by a physical rule: the larger-diameter shell contains more atoms per projected length and therefore produces deeper wall fringes and higher-intensity layer lines, so the larger measured diameter was paired with the stronger layer-line triplet. Each (diameter, angle) pair was then assigned (n, m) with the same geometry lookup used for SWCNTs.

### 7. Robustness analysis by simulation

In addition to the experimental repeatability test, robustness was assessed by simulation to consider the influence from electron dose (shot noise), camera pixel size, and field of view (tube length). Dose and pixel size are detector effects, so they were applied as CPU post-processing to one clean simulation per tube. Tube length changes the specimen model (atom count, ends, number of lattice periods) and so requires a fresh multislice per length on the GPU. The test set was the single family (20,1)–(20,19), which sweeps the chiral angle from 2.42° to 29.15° and the diameter from 1.61 to 2.65 nm. Every image was analyzed by the existing pipeline without modification — automatic region-of-interest selection, wall-projection diameter (d_ave mode), FFT with center finding, central-area masking and background subtraction, Lorentzian layer-line fitting, D-value assignment, and geometry lookup.

One clean image per tube was produced by frozen-phonon multislice (8 configurations, RMS displacement σ = 0.1 Å, plane-wave illumination, exit wave then combined with the CTF) at 60 kV, C_s = 1 µm, C_c = 1 mm, energy spread 0.35 eV and objective semi-angle cutoff 45 mrad, for a 30 nm tube in a 32 nm supercell at 0.0127 nm/px (2520 × 2520 px). Images were formed at +4 nm defocus (underfocus) referenced to the tube-center plane.

To simulate the influence from electron dose, Poisson shot noise was applied to the clean image. For a dose D (e/Å²) and pixel size p (nm) the counts per pixel are N = D·(10p)², drawn from a Poisson distribution, so the signal-to-noise ratio scales as √N. Doses from 10 to 10 000 e/Å² were tested at 0.05 nm/px; for reference, standard HRTEM imaging uses of order 500–2000 e/Å², low-dose imaging tens to a hundred, and ultra-low-dose work about 8. To simulate the influence of camera pixel size, a coarser camera was modelled by area-weighted, anti-aliased down-sampling of the fine clean image to the target pixel size. Ten pixel-sizes from 0.0127 to 0.10 nm were tested. The influence of field of view was simulated with nanotubes of different lengths. Each length was re-simulated, with all parameters unchanged, except for the length. Five lengths from 5 to 30 nm were tested at 0.05 nm/px.

## Supporting figures

**Figure S1.** Architecture of the “VibeTube” code for extracting CNTs’ chirality from TEM images.

**Figure S2.** CNT supercell for TEM image simulation. The supercell is a cube with a size of 32 nm, with the nanotube at the middle, which defines the reference level of defocus. The lengths of nanotubes are fixed at 30 nm for all tubes, regardless of the size of the unit cell, which depends closely on the chiral indices (n, m). Nanotube axis is along x direction and perpendicular to y direction. The electron beam propagates along z direction.

**Figure S3.** Diameter measurement by using d_min at under and over focus conditions. (a-b) simulated TEM images of (20, 10) CNT at under and over focus conditions, with df at 4 nm and -4 nm, respectively. (c-d) Projected intensity profile, detected max and min positions corresponding to the CNT wall fringes, and diameters measured by using d_min method.

**Figure S4.** Processing FFT for simulated (20, 10) CNT with defocus at +4 nm. (a-b) and (c-d) show the FFT in full size and in the central area, respectively. The raw FFT is plotted in logarithmic scale (a, c). After masking the central area with a square of 6.5 nm^-1^, and subtracting the background gradients by Gaussian method, the layer lines appear clearly in the central area (b, d).

**Figure S5.** Processing FFT for simulated (20, 10) CNT with defocus at -4 nm. (a-b) and (c-d) show the FFT in full size and in the central area, respectively. The raw FFTs are plotted in logarithmic scale (a, c). After masking the central area with a square of 6.5 nm^-1^, and subtracting the background gradients by Gaussian method, the layer lines appear clearly in the central area (b, d).

**Figure S6.** Layer line detection and measurement of the FFT from (20, 10) CNT at an overfocus condition. (a) Projection profile of the processed FFT with multiple peaks detected. (b) Fitting and peak detection in D1-D2 region by using a pseudo-Voigt method. (c) Fitting in D3 region by using the symmetric algorithm. (d) FFT in the central area with detected peak positions marked, corresponding to the peaks in (a).

**Figure S7.** Error analysis for chirality extraction from simulated dataset. (a) Error rate as a function of defocus. (b) Error rate as a function of tube diameter. (c) Error rate as a function of chiral angle. (d) Error rate as a function of chiral indices (n, m).

**Figure S8.** Error analysis for chirality extraction from simulated dataset with diameter measured by using d_min mode. (a) Error rate as a function of defocus. (b) Error rate as a function of tube diameter. (c) Error rate as a function of chiral angle. (d) Error rate as a function of chiral indices (n, m).

**Figure S9.** Error analysis for chirality extraction from simulated dataset with diameter measured by using d_min mode and excluding |df|<2 nm. (a) Error rate as a function of defocus. (b) Error rate as a function of tube diameter. (c) Error rate as a function of chiral angle. (d) Error rate as a function of chiral indices (n, m).

**Figure S10.** Chirality analysis of a double-walled CNT (10,5)@(20,3) from a simulated TEM image (60 kV, 0.0127 nm/px). (a) Atomic-model cross-section (beam along z). (b) Simulated TEM image (df = +4 nm). (c) Four dark-wall minima grouped by depth: the two deepest (red) span the larger shell (1.73 nm), the two shallowest (blue) the smaller shell (1.07 nm). (d) FFT of the image. (e) Six FFT layer lines (two per D-set); the two triplets, paired by D3 = D1 − D2, give chiral angles of 6.9° and 19.3°. (f) Pairing the larger diameter with the stronger layer-line triplet assigns (20,3) and (10,5), matching the isolated single-wall references.

**Figure S11.** Chirality analysis of a (10,5)+(20,3) bundle across three projected-overlap regimes. (a–c) Simulated TEM images for full overlap (Δy = 0), partial overlap (Δy = 0.85 nm) and no overlap (Δy = 1.6 nm). (d) Depth-grouped wall profile (no-overlap case) giving the two diameters. (e) FFT layer-line projection giving the two chiral angles. (f) (n,m) assignment per regime: full and no-overlap recover (20,3)+(10,5) exactly; partial overlap gives (21,3)+(10,5), the outer index shifted by one because the walls interfere in the overlap region. The chiral angles are correct in all regimes.

**Figure S12.** Robustness of the automated (n, m) assignment to acquisition conditions, for the (20,1)–(20,19) family at df = +4 nm, 60 kV, C_s = 1 µm. (a) Accuracy vs electron dose at 0.05 nm/px. Shaded bands mark standard-HRTEM (500–2000 e/Å²) and low-dose (10–100 e/Å²) regimes. (b) Accuracy vs pixel size. The diameter is over-estimated as the walls are under-sampled, resulting error in chirality identification. (c) Accuracy vs simulated tube length at 0.05 nm/px. (d, e) Accuracy maps for dose × pixel size and dose × tube length. Pixel size limits the diameter measurement precision, tube length limits the chiral angle precision, and electron dose affects both.
